# Pain and Tooth Movement During Orthodontic Leveling and Alignment—A Questionnaire-Based Study

**DOI:** 10.3390/jcm14072524

**Published:** 2025-04-07

**Authors:** Eryk Prajwos, Maciej Jedliński, Małgorzata Król, Michał Kaczmarek, Alicja Grabarczyk, Patrycja Kaźmierczak, Joanna Janiszewska-Olszowska

**Affiliations:** 1 Student Scientific Society at the Department of Interdisciplinary Dentistry, Pomeranian Medical University in Szczecin, al. Powstańców Wlkp. 72, 70-111 Szczecin, Poland; prajwoseryk@gmail.com (E.P.); michalk427@gmail.com (M.K.); alicja_grabarczyk@wp.pl (A.G.); kazmierczak.patrycja2000@gmail.com (P.K.); 2 Department of Interdisciplinary Dentistry, Pomeranian Medical University in Szczecin, al. Powstańców Wlkp. 72, 70-111 Szczecin, Poland; jjo@pum.edu.pl; 3 Department of Biochemistry, Pomeranian Medical University in Szczecin, al. Powstańców Wlkp. 72, 70-111 Szczecin, Poland; malgorzatakrol246@gmail.com

**Keywords:** orthodontic treatment, pain, tooth movement, fixed appliances, pain dynamics, external pain factors, patient experience

## Abstract

**Background:** Orthodontic treatment with fixed appliances often induces pain. Despite existing research on pain management, the dynamic nature of orthodontic pain and its relationship with tooth movement remain underexplored. This study surveys adults under fixed appliance therapy to investigate pain dynamics, modifying factors, and perceived tooth movement, aiming to provide insights for improved patient care and treatment outcomes. **Methods**: This study focused on healthy individuals aged 18 to 50 undergoing fixed appliance treatment for up to six months after having braces bonded. A 24-question survey was administered over a one-month period. The survey explored pain intensity, pain dynamics, modifying factors, and perceived tooth movement. **Results**: Pain typically began within the first few hours after bonding and peaked the following day for most respondents. The upper incisors and molars were the most frequently reported areas of pain, corresponding to the teeth perceived as moving earliest. Chewing was identified as the primary external pain trigger. Gender significantly influenced pain perception, with women reporting higher pain levels and greater use of medication. The majority of participants managed without medication. **Conclusions**: Pain during the early phase of fixed appliance therapy follows a predictable pattern, with peak intensity occurring on the day after bonding. Pain perception strongly correlates with early tooth movement, particularly in the upper incisors. Gender differences were evident, but other demographic factors had minimal influence. Chewing was the primary pain aggravator, emphasizing the need for dietary modifications. Understanding these findings can help orthodontists develop personalized pain management strategies.

## 1. Introduction

Orthodontic treatment with fixed appliances is widely used to correct malocclusion and improve dental aesthetics and function [1]. Tooth movement involves controlled mechanical forces, stimulating remodeling of the periodontal ligament (PDL) and alveolar bone [1,2]. While effective, this process often induces varying degrees of pain and discomfort, which are significant concerns for patients and can impact their compliance with treatment [3].

Pain during orthodontic therapy is multifactorial, influenced by individual pain tolerance, the type and intensity of forces applied, and psychosocial factors. Current literature highlights several key contributors to pain perception in patients with fixed appliances. These include the timing of orthodontic activation, the duration since appliance bonding, and external stimuli such as chewing or temperature changes [4,5]. Additionally, demographic factors such as gender, age, place of residence, and educational level can play a role [4]. Psychological aspects, including pain catastrophizing and anxiety, further influence pain perception [6]. Genetics may also affect the perception of the pain, as variations such as COMT gene polymorphisms and HTR2A and NR3C1 gene variants have been linked to differences in pain sensitivity [7]. Studies indicate that 87% to 95% of patients experience pain during fixed orthodontic treatment, particularly within the first 24 h. Moreover, 39% to 49% report discomfort at each stage of treatment or after appliance removal [8,9].

Orthodontic pain typically arises from the inflammatory response of periodontal tissues to mechanical forces exerted by orthodontic appliances [10], especially throughout the initial stages of treatment, appliance adjustments, and activation [3]. The current state of knowledge on pain after orthodontic bonding indicates that pain typically begins shortly after appliance insertion, with an average onset around 4 h. Pain peaks within the first 24 h, averaging 42.4 mm (millimeters) on the visual analogue scale (VAS), and then gradually declines, reaching around 9.0 mm by the seventh day. Factors influencing pain include the time of day, with higher levels in the morning compared to the evening, and activities such as chewing, which significantly increase discomfort. About 62% of patients use analgesics within the first 6 h, with usage declining to less than 10% by the seventh day. While gender, age, or tooth irregularity do not consistently affect pain levels, patients undergoing tooth extractions report notably higher pain, particularly in the mandible. An average initial pain score of 12.9 mm was reported immediately after appliance bonding, with maximum pain peaking at 68.2 mm. By the end of the first week, pain levels decreased to an average of 9.0 mm. Analgesic use was common, with around 54% of patients taking pain relief at least once during the first week, often in anticipation of discomfort [11]. Understanding the dynamics of this pain and its relationship with tooth movement is critical for improving patient care and enhancing the overall treatment experience.

Although several studies have investigated orthodontic pain, many have focused on specific aspects, such as pharmacological management or pain thresholds. Few have comprehensively explored the dynamic nature of pain and the perceived onset of tooth movement during the initial phase of treatment. Additionally, the influence of demographic, environmental, and psychological factors on pain perception and treatment experience remains underexplored.

Thus, this study seeks to address these gaps by surveying adult patients undergoing fixed appliance therapy. It investigates the dynamics of pain, factors influencing its intensity and duration, the impact of external stimuli, and patients’ perception of tooth movement during the first six months of treatment. By identifying patterns and correlations, this study seeks to provide insights that can guide clinicians in managing patient discomfort and optimizing treatment outcomes. The null hypothesis is that none of these factors influence pain perception.

## 2. Materials and Methods

### 2.1. Sample Size Adjustment

Sample size adjustment was performed at the level of significance α = 0.05 using the following online calculator—SurveyMonkey, San Mateo, CA, USA [12]. A total of 60 patients represented 20% of those admitted to the orthodontic clinic during the one-month study period.

### 2.2. Ethical Approval of the Survey and Administration of the Survey

This questionnaire study was partially retrospective and was exempted from approval by the bioethical committee of Pomeranian Medical University with decision reference number KB.006.001/2025 [Supplementary Material S1]. The target population was healthy adults between 18 and 50 years of age. Patients with any chronic diseases or psychoemotional disorders were excluded from this study. The survey was carried out in Polish within a month, from 22 January 2025 to 22 February 2025. The questionnaire consisted of 24 questions and was designed using Google Forms (Google, Mountain View, CA, USA). The questionnaire was conducted with patients immediately following their scheduled follow-up visits at an orthodontic clinic in Koszalin (Poland). The full Polish version of the questionnaire is available as Supplementary Material [Supplementary Material S2]. 

### 2.3. Statistical Analysis

Pearson’s chi-squared test was used to compare different response categories between patient groups. For comparing the means of the continuous variable (maximum pain) across groups, a *t*-test was applied. Spearman’s correlation coefficient was used to assess the association between ordinal variables. A series of linear regression models were constructed to investigate the influence of individual patient characteristics on reported maximum pain intensity. The optimal model was selected based on the Akaike Information Criterion (AIC) [13]. Statistical significance was determined at a threshold of *p* < 0.05. All analyses were conducted using the R statistical software [14].

## 3. Results

### 3.1. Sample Size Adjustment

The sample size was estimated as at least 50 people at a level of significance of α = 0.05.

### 3.2. Demographic Characteristics of the Respondents

#### 3.2.1. Age

**Figure 1:**
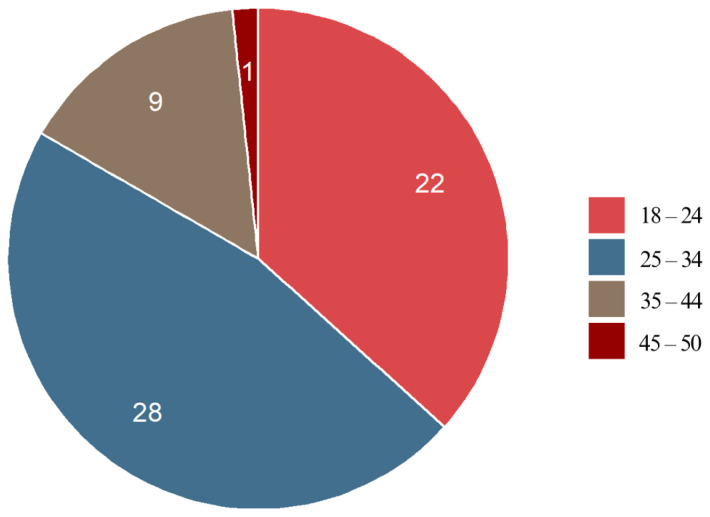
Age characteristics of the respondents.

The majority of the respondents (83.4%) were between 18 and 34 years old, suggesting that young adults are the primary group undergoing fixed appliance therapy. The highest representation was in the 25–34 age range, indicating that individuals in this group may be more likely to seek orthodontic treatment. In contrast, only one respondent (1.7%) was aged 45–50, and none was over 50, highlighting that fixed appliance therapy is less common among elderly adults (Figure 1).

#### 3.2.2. Gender

**Figure 2:**
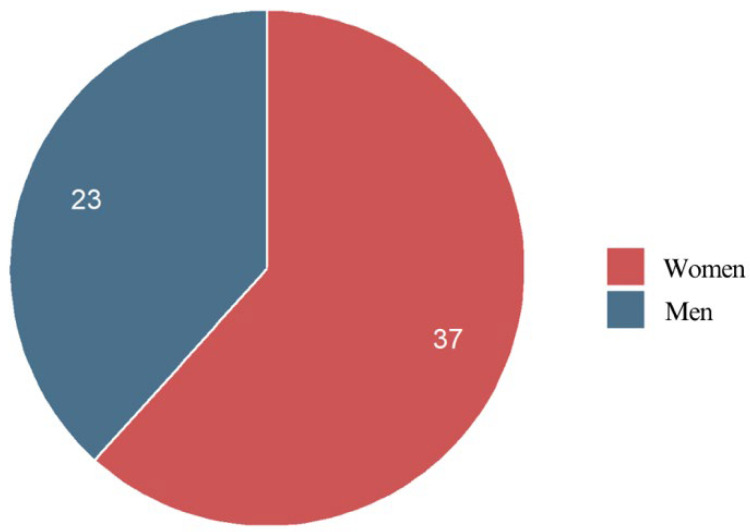
Gender characteristics of the respondents.

The gender distribution of the respondents shows that females comprised the majority of the participants. Specifically, 37 out of 60 respondents (61.7%) were female, while 23 (38.3%) were male. This suggests that women may be more likely to seek orthodontic treatment with fixed appliances or may be more willing to participate in questionnaire-based research on orthodontic experiences (Figure 2).

#### 3.2.3. Place of Residence

**Figure 3:**
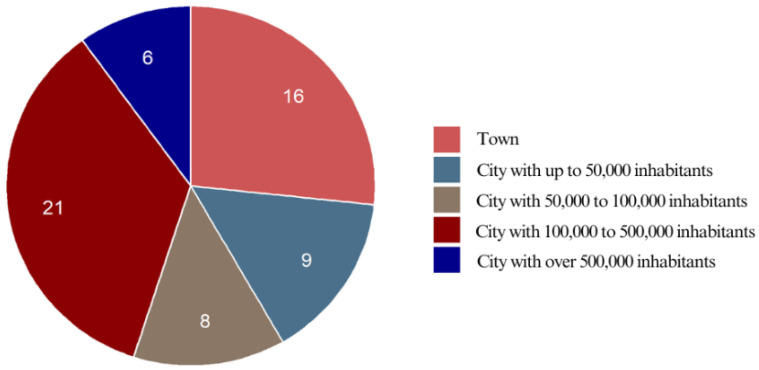
Place of residence referring to number of respondents.

The majority of the respondents (73.3%) resided in urban areas, with the largest proportion (35.0%) living in cities with 100,000–500,000 inhabitants. More than a quarter (26.7%) came from rural areas, indicating that fixed appliance therapy is sought by individuals from both urban and rural settings. Only 10.0% of the respondents lived in cities with over 500,000 inhabitants, suggesting that the sample was not heavily skewed toward large metropolitan centers. The distribution across small and medium-sized towns (up to 100,000 inhabitants) was relatively balanced, accounting for 28.3% of the total (Figure 3).

#### 3.2.4. Educational Level

**Figure 4:**
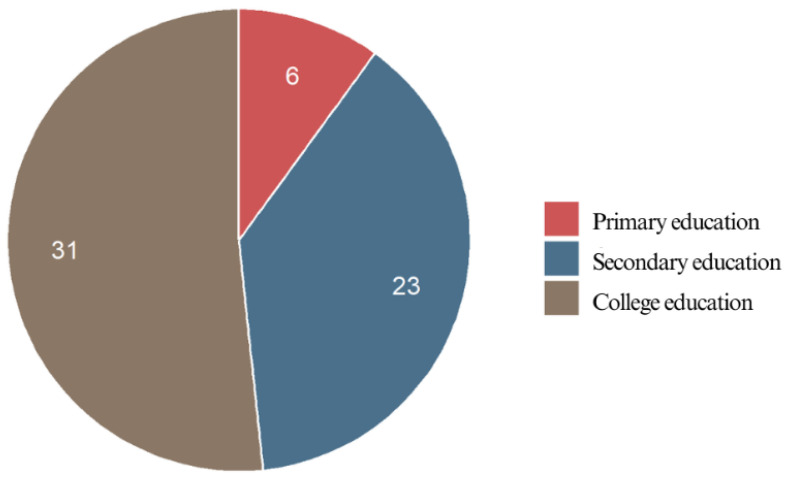
Educational level of the respondents.

A significant proportion of the respondents (51.7%) held a higher education degree, indicating that individuals with advanced education levels were well-represented in this study. Nearly two-fifths (38.3%) had completed secondary education, making it the second-largest group. Meanwhile, only 10.0% had only a primary education, suggesting that this study predominantly included individuals with at least a secondary-level education (Figure 4).

### 3.3. Orthodontic Treatment Details

#### 3.3.1. Duration of Orthodontic Treatment

**Figure 5:**
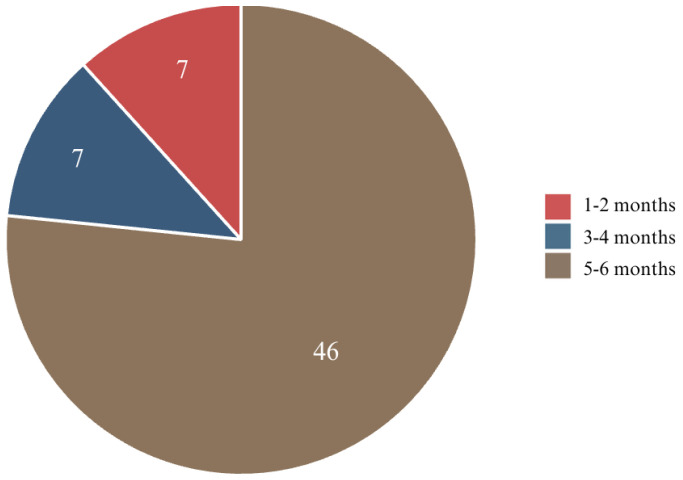
Duration of orthodontic treatment with fixed appliances.

The analysis of the treatment duration among the respondents revealed that the majority (76.7%) had been wearing fixed braces for 5–6 months. A smaller proportion (11.7%) had their braces for 3–4 months, while an equal percentage (11.7%) had them for only 1–2 months. These findings indicate that most of the participants were in the initial phase of their orthodontic treatment, which is typically when significant tooth movement and associated discomfort occur (Figure 5).

#### 3.3.2. Braces Placement

**Figure 6:**
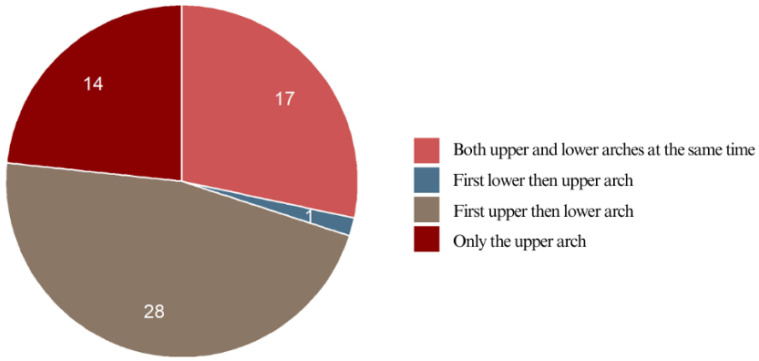
Braces placement of the respondents.

The majority of the respondents (46.7%) had their upper teeth bonded first, followed by their lower teeth. In contrast, only 1.7% experienced the reverse sequence, with braces bonded to the lower teeth first, whereas 28.3% had braces placed on both arches simultaneously. Another 23.3% received braces only on the upper teeth, making this the second most common approach after “upper first, then lower”. Notably, none of the respondents had braces placed solely on their lower teeth. Simultaneous treatment (28.3%) was less frequent than phased placement, while the least common approach was starting with the lower teeth (1.7%) (Figure 6).

### 3.4. Pain Experience

#### 3.4.1. Maximum Pain Level

Pain experience was reported by all the patients. The average reported pain intensity was 46.58 out of 100. The most frequently reported pain level was 50, reported by 18.3% of the participants. Other commonly reported pain levels included 40 (11.7%), 60 (10.0%), 30 (10.0%), and 90 (8.3%). Only one respondent (1.7%) reported the maximum pain level of 100. While some participants experienced minimal pain—1.7% rated it at 1 or 3 and 3.3% at 5 or 25—the majority (68.3%) reported peak pain levels between 30 and 90, suggesting that moderate to severe pain was typical. Conversely, only 8.4% of the respondents reported very mild pain (1–10), indicating that discomfort after bonding braces is a common experience.

#### 3.4.2. Pain Onset

**Figure 7:**
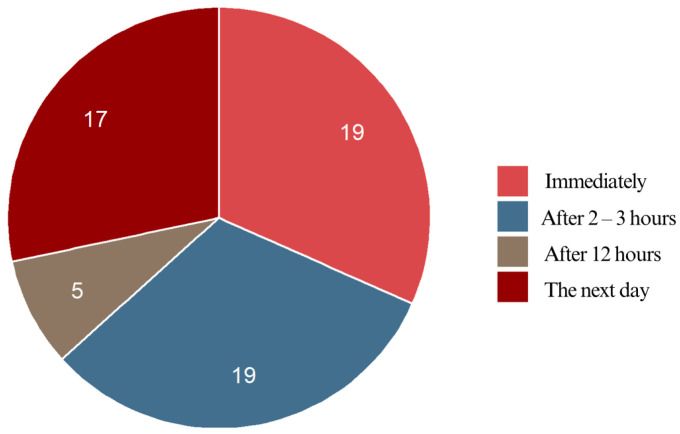
Pain onset of the respondents.

Pain onset varied among the respondents, though most reported discomfort within the first few hours after bonding. After bonding, 31.7% of the respondents felt pain right away, while another 31.7% experienced it within 2–3 h. A smaller group (8.3%) reported pain beginning around 12 h later, whereas 28.3% felt discomfort only the next day upon waking. Overall, 63.4% of the respondents experienced pain either immediately or within a few hours, indicating that early discomfort is a common response to braces bonding. In contrast, 36.6% reported a delayed onset, with 28.3% feeling pain the next day and 8.3% after 12 h. The data highlight two key peaks in pain onset: the first occurring immediately or within a few hours (63.4%), and the second occurring the following day (28.3%). This suggests that while pain typically starts soon after bonding, for some individuals, it develops more steadily (Figure 7).

#### 3.4.3. Pain Intensity Spike

**Figure 8:**
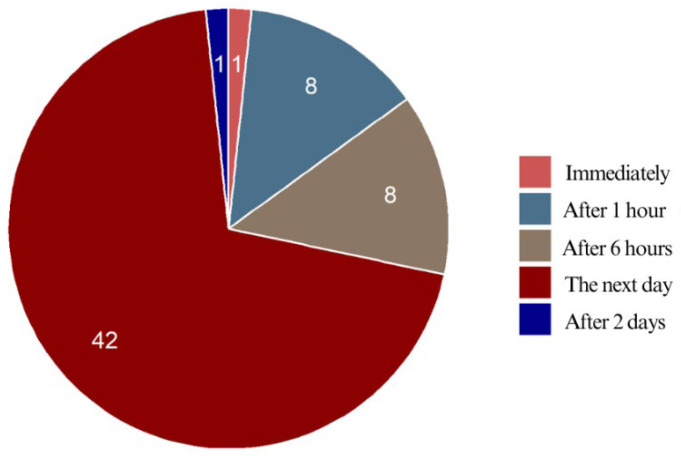
Peak pain intensity of the respondents.

Pain intensity increased gradually over time, with most of the respondents experiencing the worst pain the day after bonding. Only 1.7% reported peak pain occurring right away, while 13.3% felt the most intense pain after 1 h, and another 13.3% after 6 h. The majority (70.0%) experienced their highest pain intensity the following day, while a small percentage (1.7%) reported peak pain on the second day. More than 70% of the respondents experienced the most intense pain the day after bonding. Moreover, 28.3% reported peak pain occurring within the first few hours to the first day, highlighting individual differences in pain perception (Figure 8).

#### 3.4.4. Pain Characteristics

A diverse range of pain characteristics was reported, with some of the respondents experiencing a gradual decrease in discomfort, while others reported persistent or increasing pain. A third of the respondents reported that their pain gradually diminished over time, while another third experienced persistent but low-intensity pain. Additionally, 28.3% noted that their pain initially intensified before eventually subsiding, while 21.7% experienced ongoing, high-intensity pain. Additionally, 11.7% described their pain as sudden in onset, while 1.7% reported that it disappeared abruptly. A small fraction of the respondents (3.4%) provided more specific descriptions of their pain: 1.7% experienced a pulling sensation between their teeth, lasting only a day after wire replacement, and the other 1.7% reported pain occurring specifically while eating.

The most commonly reported pain experiences were gradually decreasing discomfort (33.3%) and constant mild pain (33.3%), indicating that many of the respondents adapted to the discomfort over time. More than half (55%) reported persistent pain, either at low intensity (33.3%) or high intensity (21.7%), suggesting that for some, discomfort remains steady rather than fluctuating. A notable portion of the respondents, 28.3%, experienced increasing pain over time. Less common pain patterns included a sudden onset of pain, reported by 11.7%, and pain that disappeared abruptly, noted by 1.7%. These findings highlight the individual variability in pain perception.

#### 3.4.5. Impact of External Stimuli

**Figure 9:**
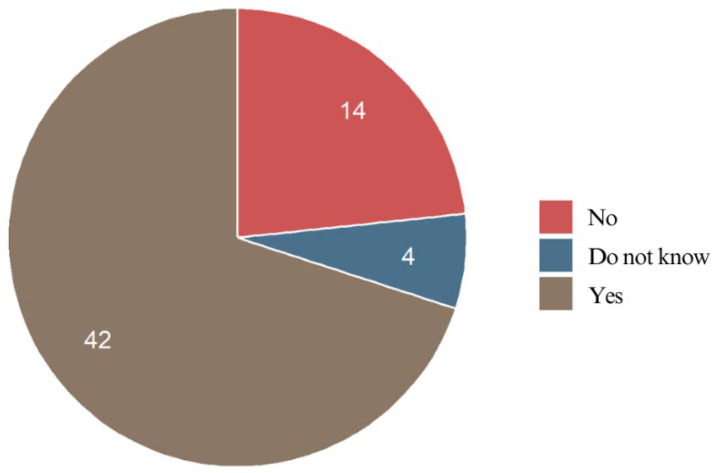
Influence of external stimuli on respondents’ pain perception.

The majority (70.0%) of the respondents reported that chewing, temperature changes, or pressure intensified their pain. Nearly one-fourth (23.3%) did not experience any increase in pain, highlighting variability in individual pain sensitivity. A small percentage (6.7%) were unsure of whether external stimuli influenced their pain, possibly due to subtle or inconsistent pain responses (Figure 9).

#### 3.4.6. Common Pain Triggers

Chewing was the primary pain trigger, affecting 70.0% of the respondents. This aligns with the mechanical pressure exerted on teeth during orthodontic movement, which increases sensitivity and discomfort. In contrast, temperature-related stimuli had a lesser impact, with cold (10.0%) being slightly more aggravating than heat (8.3%). This may be due to the teeth’s thermal sensitivity as they adjust to orthodontic forces.

#### 3.4.7. Tooth Pain Patterns

The tooth pain patterns among the respondents are presented in Table 1.

The upper incisors were the most commonly reported as painful. Upper premolars and molars also caused significant pain, affecting almost half of the participants. In contrast, lower teeth were less frequently associated with pain, with the lower incisors being the most affected, followed by lower premolars and molars. Overall, pain levels varied by tooth position, with the most discomfort occurring in the upper incisors and molars, while lower teeth and canines were less affected.

#### 3.4.8. Impact of Gender on Pain

No significant relationship was found between genders and the onset of pain (Pearson’s Chi-squared test, *p* = 0.356) or the time at which pain became most intense (*p* = 0.303), suggesting that men and women experienced similar pain timing patterns. However, a difference in pain levels between genders (*t*-test, *p* = 0.011) showed that women reported higher average pain scores (52.68 ± 25.89) compared to men (36.78 ± 20.57). These findings indicate that while the timing of pain onset and peak intensity does not appear to be influenced by gender, women tend to experience higher overall pain levels during the initial phase of orthodontic treatment.

#### 3.4.9. Impact of Age on Pain

Younger patients (18–24 years) reported slightly higher pain levels (Mean = 48.18) than older patients (Mean = 45.66) (*p* = 0.678). When the younger group was expanded to include patients aged 18–34, their reported pain levels (Mean = 44.1) were lower than those of the older group (Mean = 59.0) (*p* = 0.133). These findings suggest that age does not have a significant impact on reported pain levels after bonding braces. While some variations exist, they are not substantial enough to indicate a consistent trend in pain perception between younger and older patients.

#### 3.4.10. Impact of Place of Residence on Pain

Patients from larger cities (>50 k and >100 k population) reported slightly higher pain levels (49.54 and 49.07) compared to those from smaller towns (<50 k and <100 k), with mean pain levels of 42.44 and 44.55. However, these differences were not statistically significant (*p* = 0.273 and *p* = 0.488) and may have been due to random chance. The high standard deviations suggest considerable variability in reported pain levels, indicating that city size does not have a clear impact on pain perception, despite a slight trend of higher pain in larger cities.

#### 3.4.11. Impact of Simultaneous vs. Nonsimultaneous Treatment on Pain

The analysis examined whether patients who had both arches treated simultaneously reported higher pain levels compared to those who had their braces applied to one arch at a time. The results showed that patients with simultaneous treatment reported a slightly higher average pain level (Mean = 52.06) compared to those who had staged treatment (mean = 44.42) (*p* = 0.315).

#### 3.4.12. Relationship Between Treatment Time and Pain Intensity

No significant relationship between the duration of wearing braces and the intensity of pain experienced over time was found. This finding implies that while some patients may experience reduced discomfort over time, others may continue to report similar levels of pain regardless of how long they have had their braces. Pain perception is likely influenced by individual sensitivity, orthodontic adjustments, and biological factors rather than just treatment duration.

#### 3.4.13. Impact of External Stimuli on Pain

Patients who experienced increased pain when biting reported a maximal pain level of 49.29 (SD = 26.14) (standard deviation), compared to 40.28 (SD = 21.66) in those without biting sensitivity. Similarly, those affected by temperature changes reported an average pain level of 54.33 (SD = 34.42), while those unaffected had a mean of 45.72 (SD = 24.07). However, neither biting sensitivity (*p* = 0.174) nor temperature-related pain (p = 0.574) showed a statistically significant correlation with higher initial pain levels (*p* > 0.05). These findings suggest that external stimuli do not play a major role in determining pain intensity. Instead, factors such as individual pain thresholds or orthodontic treatment specifics may have a greater impact on pain perception.

#### 3.4.14. Impact of Educational Level on Pain

The comparison of reported pain levels between patients with higher education and those with other education levels showed that patients with higher education reported a mean pain level of 49.68 (SD = 29.09), while those with other education levels had a mean pain level of 43.28 (SD = 19.83) (*p* = 0.321). This suggests that education level does not significantly influence perceived pain during the initial phase of fixed appliance therapy. Instead, factors such as individual pain tolerance, anxiety levels, and biological responses may play a more substantial role in pain perception.

#### 3.4.15. Relationship Between Educational Level and Pain Medication Use

No clear trend regarding the relationship between education level and the likelihood of taking pain medication during the initial phase of fixed appliance therapy was found. Patients with higher education (64.5%) were slightly more likely to report not taking pain medication compared to those with secondary education (73.9%) and primary education (83.3%) (*p* = 0.571). Conversely, the proportion of patients who took pain medication was somewhat higher among those with higher education (35.5%) than among those with secondary (26.1%) or primary education (16.7%).

#### 3.4.16. Relationship Between Educational Level and Tooth Movement Perception

Patients with secondary education (60.9%) reported noticing immediate tooth movement slightly more often than those with a college education (51.6%) and primary education (16.7%). However, there was no clear trend or statistically significant pattern indicating that a higher level of education is associated with a greater likelihood of perceiving early tooth movement (*p* = 0.281). Additionally, a notable proportion of patients in all education groups reported uncertainty about whether their teeth were moving (primary: 66.7%; secondary: 21.7%; college: 29.0%).

### 3.5. Pain Management

#### 3.5.1. Medication Usage

**Figure 10:**
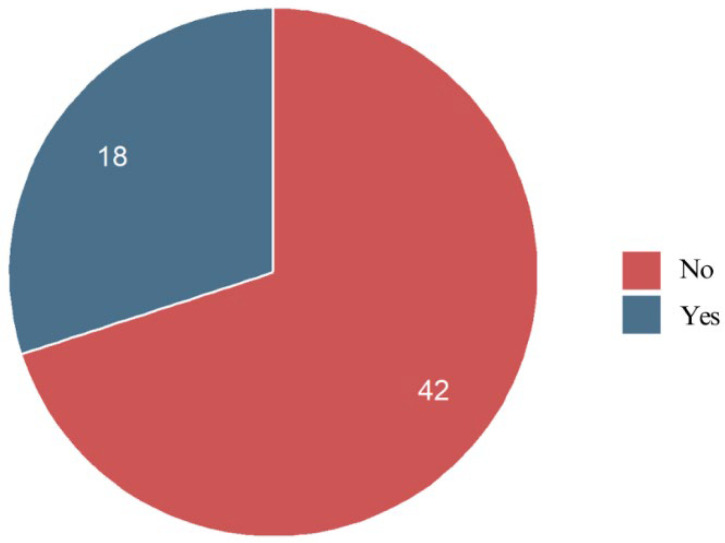
Pain medication usage of the respondents.

The majority of the respondents (70%) managed their pain without medication, suggesting that their discomfort was either mild or they adapted to it over time. In contrast, 30% used pain relief, indicating that for some, the pain was intense enough to require medication. These findings highlight the variability in pain experiences after braces bonding, with most patients experiencing mild to moderate discomfort, while a smaller group required medication to manage more significant pain (Figure 10).

#### 3.5.2. Pain Medication Preferences

The data revealed that ibuprofen was the most used pain reliever, taken by 47.6% of the respondents, indicating its widespread preference for managing discomfort after braces bonding. Paracetamol was the second most frequently used medication, reported by 23.8% of the respondents, suggesting its role as an alternative for pain relief. Ketoprofen and nimesulide were each used by 14.3% of the respondents, likely chosen for their stronger anti-inflammatory properties. These findings indicate that ibuprofen was the preferred pain management option, often used alone or in combination with other medications. Stronger pain relievers like ketoprofen and nimesulide were used by a smaller group, likely reflecting higher pain levels in those individuals.

#### 3.5.3. Impact of Gender on Medication Usage

A significant difference in medication usage between genders was found, with women being more likely than men to take pain medication after braces bonding. Among the female respondents, 40.5% used pain relievers, compared to just 13.0% of the male respondents (*p* = 0.0488). These findings suggest that women may experience greater discomfort or have a lower pain tolerance, resulting in a higher reliance on pain relief.

#### 3.5.4. Impact of Medication Usage on Pain Level

Painkiller users reported higher average pain levels (57.28 ± 24.02) compared to those who did not take medication (42.0 ± 24.33) (*p* = 0.031). These findings suggest that individuals experiencing more intense pain were more likely to seek relief through medication, highlighting the variability in pain perception and tolerance among orthodontic patients.

### 3.6. Tooth Movement Perception

#### 3.6.1. Perception of Immediate Tooth Movement

**Figure 11:**
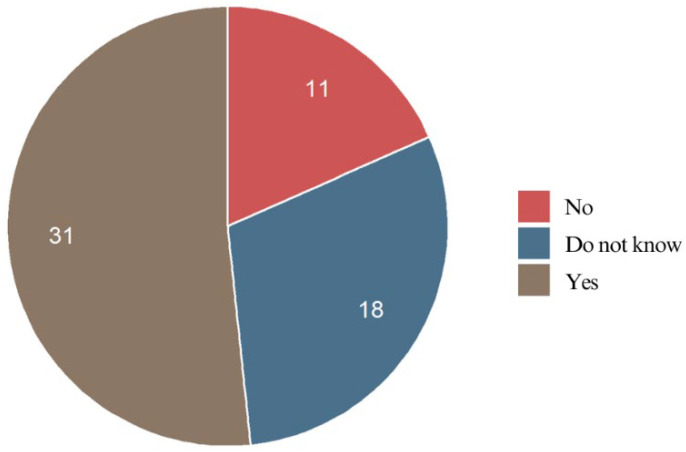
Pain perception of the respondents.

More than half of the respondents (51.7%) believed that their teeth started moving immediately after bonding braces. Moreover, patients notice a certain relationship between pain and orthodontic tooth movement. A significant portion (30.0%) were unsure, indicating that tooth movement may not always be consciously felt in the initial phase. Meanwhile, 18.3% did not believe their teeth started moving right away (Figure 11).

#### 3.6.2. Teeth Most Affected by Initial Tooth Movement

Upper and lower incisors were the first teeth to begin moving after braces bonding. In the upper dental arch, 63.3% of the respondents reported initial movement of the incisors. Premolars and canines were indicated as the first teeth moving by 21.7% of the respondents, while molars were the least likely to shift early (10.0%). For lower teeth, incisors were again the most frequently reported as the first to move, with 73.9% of the respondents noticing changes in this region. Canines were perceived as the first to move by 19.6% and premolars by 17.4%, while molars (6.5%) were most rarely reported as the first teeth to move. These findings may suggest that incisors, particularly in the lower arch, respond most quickly to orthodontic forces, while molars tend to shift more gradually.

#### 3.6.3. Impact of Age on Tooth Movement Perception

The distribution of responses was fairly consistent across age groups, suggesting that age does not significantly influence perceived tooth movement after bonding braces (*p* = 0.879).

#### 3.6.4. Relationship Between Tooth Movement Perception and Most Painful Teeth

The patients frequently reported pain in their upper incisors, premolars, and molars, while they perceived the incisors and premolars as moving first. Similarly, in the lower jaw, incisors, canines, and premolars were most often reported as painful, with incisors and canines perceived as the first to shift. This strong correlation suggests that the patients tended to experience early tooth movement in the same areas where pain was most intense. Pain may serve as a predictor of initial tooth displacement during the early phase of fixed appliance therapy. The highest discomfort and earliest movement typically occurred in the anterior teeth and premolars.

#### 3.6.5. Relationship Between Braces Placement and Tooth Movement Perception

Simultaneous treatment of both arches did not significantly influence the patients’ perception of early tooth movement. While a slightly higher proportion of the patients in this group reported immediate movement (58.8%) compared to those treated non-simultaneously (48.8%), the difference was not statistically meaningful. Treating only the upper teeth did not lead to a consistently faster perception of movement compared to other approaches (*p* = 0.368). Sequential treatment showed no significant impact on perceived movement speed (*p* = 0.196). These findings suggest that treatment sequencing or arch-specific protocols do not strongly influence how quickly patients perceive tooth movement. Instead, factors such as pain sensitivity, biological response, and force application may play a more significant role.

### 3.7. Regression Analysis

To determine which patient characteristics were associated with maximum pain perception, multiple linear regression models were evaluated. Predictor variables included gender, age (with two smaller older-age categories combined into a single 35–50 group), educational background, presence of chronic diseases, and medication usage after orthodontic braces bonding and/or on a regular basis. Interaction terms were also considered. The results of the model estimation are presented in Table 2.

The final model was statistically significant (F [5, 54] = 4.5, *p* = 0.002) and explained almost 30% of the between-patient variance in the maximum pain level. The intercept corresponded to the expected pain score for a reference patient: a female aged 18–24 years, with no chronic diseases and not taking any medications following appliance placement.

Patients aged 25–34 reported significantly lower pain scores compared to the reference group. Conversely, individuals aged 35–50 exhibited higher pain levels, although this difference did not reach statistical significance—possibly due to the limited sample size in this subgroup (n = 10). A statistically significant increase in reported pain was observed among the patients who took medications following braces bonding. While male patients showed trends towards lower pain and those with chronic diseases towards higher pain, these associations were not statistically significant.

## 4. Discussion

Orthodontic pain arises when mechanical forces applied during treatment trigger biological responses in the PDL, alveolar bone, and surrounding tissues. This pain is primarily inflammatory and occurs in two phases. During the acute phase (first 24–72 h), ischemia, the release of inflammatory mediators, and nociceptor activation lead to pain and discomfort. This is followed by the adaptation phase (3–7 days), during which osteoclasts resorb bone on the compression side while osteoblasts deposit new bone on the tension side, resulting in a gradual reduction in pain [15]. However, excessive orthodontic forces may cause undermining necrosis, leading to vascular occlusion, tissue damage, and a prolonged inflammatory response. This, in turn, can delay normal bone remodeling and cause persistent pain due to delayed osteoclastic activity and extended tissue breakdown [16,17,18].

The findings of this study confirm that most patients experience peak pain within 24 h of bonding, followed by a gradual decline over the first week. Chewing was identified as the primary aggravating factor, while temperature changes had a lesser impact. A strong correlation was observed between the location of pain and the perceived onset of tooth movement, particularly in the upper incisors and molars. However, no suitable studies were found to support this correlation. These findings suggest that discomfort may serve as an early indicator of orthodontic force application. While many patients adapted to the discomfort over time, a significant number continued to experience moderate to severe pain, highlighting the need for personalized pain management strategies.

The predominance of younger adults, particularly those aged 18–34, with the highest representation in the 25–34 age range, suggests that individuals in this demographic may be more proactive in seeking orthodontic care. This trend likely stems from increased awareness of dental aesthetics and oral health, aligning with the findings of Sfeatcu et al. (2022) [19]. Furthermore, the higher proportion of female participants in our sample (61.7%) is consistent with previous research indicating that women are more likely to seek dental treatment. This may be due to greater aesthetic concerns, more proactive health-seeking behaviors, or a higher willingness to participate in research on orthodontic experiences [19].

Patients in this study generally reported moderate to severe pain, with the most commonly reported peak pain score being 50 on a 100-point scale and an average pain intensity of 46.58 out of 100. However, direct comparisons with existing literature were not possible, as prior studies did not use a 100-point scale. Nonetheless, these findings align with those of Inauen et al. (2023) [11], who used the VAS scale; when converted, their results correspond to a pain score of 42.4 out of 100. The onset of pain within a few hours after bonding, peaking the following day, is consistent with the inflammatory response triggered by orthodontic forces [20]. Although some responses may reflect generalized pain experiences rather than precise daily recall, these findings remain valuable, particularly given the subjective nature of pain assessment.

External factors played a notable role in pain experiences. Chewing was identified as the primary pain trigger by 70% of the respondents, while sensitivity to temperature changes was less common (reported by 10% for cold and 8.3% for heat). These findings emphasize the importance of dietary modifications—such as consuming softer foods—during the early phase of treatment. The upper incisors (58.3%) and upper molars (48.3%) were the most frequently reported sites of pain, likely due to their direct exposure to orthodontic forces. Interestingly, these teeth were also perceived as the first to move, suggesting a potential link between pain localization and early orthodontic changes. Nevertheless, no relevant studies could be found to support this relationship.

The observed gender differences in pain perception—specifically, that women reported higher pain levels and used pain medication more frequently, may suggest either greater discomfort or lower pain thresholds. However, although the t-test showed a statistically significant association of pain perception with gender, it was not confirmed in the regression analysis. Thus, the regression model did not identify gender as a statistically significant predictor of pain perception. This suggests that the intensity of pain experienced by the participants may depend on other factors. These results contrast with previous studies, in which gender was found to be a significant factor influencing pain perception [21,22,23]. Nevertheless, gender did not significantly affect the timing of pain onset or peak intensity, and no relevant studies were found for direct comparison.

Our study also found that factors such as age, place of residence, and education level did not significantly impact pain perception. While no studies were found comparing pain perception based on place of residence or education level, the influence of age has been explored in several studies. Lautenbacher et al. (2017) suggest that older patients exhibit slightly reduced pain sensitivity [23], which aligns with our findings showing that younger patients (18–24 years) reported slightly higher pain levels than older individuals. Additionally, patients from larger cities (>50,000 inhabitants) experienced slightly higher pain levels than those from smaller towns. Our study also found that individuals with higher education were marginally more likely to use pain relievers, a trend consistent with the findings of Mehuys et al. (2019) [24].

We also examined the relationship between treatment methodology and pain perception. Patients undergoing simultaneous treatment of both arches reported slightly higher average pain levels (52.06) compared to those receiving staggered treatment (44.42). Notably, this is the first study to explore such a correlation. These findings suggest that while treating both arches simultaneously may lead to slightly greater discomfort, individual pain tolerance and biological responses play a more significant role. Additionally, treatment duration did not correlate with pain intensity, indicating that pain perception is influenced more by individual sensitivity, orthodontic force application, and adjustments rather than the overall length of treatment.

Regarding tooth movement perception, more than half of the respondents (51.7%) believed their teeth began moving immediately after bonding, while 30.0% were uncertain. No relevant data were found in the existing literature. This suggests that while many patients associate the initial pressure with movement, others may not perceive any changes until later. The strong correlation between tooth pain and perceived movement—particularly in the upper and lower incisors—further supports the idea that discomfort may serve as an early indicator of tooth displacement. However, the subjective nature of this assessment underscores the need for future studies to objectively correlate pain with actual tooth movement during the leveling phase.

Medication usage patterns provided additional insights into pain management strategies. The majority of the respondents (70%) managed their pain without medication, suggesting that the discomfort was tolerable for many. Among those who did take pain relievers, ibuprofen was the most commonly used (47.6%), followed by paracetamol (23.8%), while stronger anti-inflammatory medications such as ketoprofen and nimesulide were each used by 14.3% of the patients. This pattern aligns with the findings of Olteanu et al. (2022) [25], which identified ibuprofen as the most frequently used pain medication during orthodontic treatment. Notably, those who took pain medication reported higher average pain levels (57.28 ± 24.02) compared to non-users (42.0 ± 24.33), suggesting that individuals experiencing greater discomfort were more likely to seek pharmacological relief. No relevant studies were found exploring this correlation.

One of the main limitations of this study is its reliance on self-reported data, which introduces the potential for recall bias and subjective variations in pain perception. Pain levels and tooth movement were assessed based on patient responses rather than objective clinical measurements, making it difficult to precisely correlate discomfort with actual orthodontic changes. Additionally, this study did not account for psychological factors such as anxiety or pain tolerance, which could influence reported pain intensity. The lack of relevant literature for comparison in certain aspects, such as the correlation between pain perception and tooth movement or simultaneous versus staggered treatment, further limits the findings. Lastly, as this study was conducted in a single orthodontic clinic with only Polish-speaking participants, cultural and regional factors may have influenced the results, limiting their generalizability.

## 5. Conclusions

Pain during the initial phase of fixed appliance therapy follows a predictable pattern, with peak intensity occurring the day after bonding. A strong correlation was observed between pain localization and perceived early tooth movement, particularly in the upper incisors and molars. While pain intensity was not significantly influenced by gender, other factors may play a role—women reported greater use of pain medication. Variables such as age, place of residence, and education level had minimal effects. Chewing was the most common pain trigger, whereas temperature changes played a lesser role. Based on these findings, the null hypothesis was rejected.

This study offers valuable insights into pain dynamics and perceived tooth movement, highlighting the importance of personalized pain management strategies. Future research should include clinical assessments, larger sample sizes, and longitudinal designs to further explore the relationship between pain perception and orthodontic tooth movement. It should also clarify the potential role of the presence of chronic disease in the experience of pain during orthodontic treatment.

## Figures and Tables

**Table 1:** Distribution of pain by tooth type.

Response	Number	Proportion (%)
Upper incisors	35	58.3
Upper premolars and molars	29	48.3
Lower incisors	17	28.3
Lower premolars and molars	10	16.7
Upper canines	10	16.7
Lower canines	5	8.3

**Table 2:** Results of estimation of the optimal model of the influence of individual patient characteristics on the maximum pain level.

	Estimate	S.D.	*t*.stat	*p*
(Intercept)	45.92	6.411	7.162	<0.001
Gender/male	−9.295	6.375	−1.458	0.151
Age group/25–34	−13.05	6.413	−2.035	0.047 *
Age group/35–50	12.25	8.957	1.368	0.177
Chronic diseases	13.46	7.156	1.881	0.065
Medication usage	18.62	6.997	2.661	0.010 *
R^2^	Adjusted R^2^	F [5, 54]	*p*	
0.2957	0.2305	4.5	0.002	

* statistically signifcant.

## Data Availability

The data are available from the corresponding author upon reasonable request.

## Supplementary Materials

The following supporting information can be downloaded at: https://www.mdpi.com/article/10.3390/jcm14072524/s1, Supplementary File S1: Local bioethical committee decision; Supplementary File S2: Full content of the questionnaire in Polish.

## Abbreviations

The following abbreviations are used in this manuscript:

PDL	Periodontal Ligament
VAS	Visual Analogue Scale
SD	Standard Deviation
rho	Spearman’s Rank Correlation Coefficient
S	Sum of Squared Differences
N	Sample Size
mm	Millimeters
