# Supplementary material for: Pain and Tooth Movement During Orthodontic Leveling and Alignment—A Questionnaire-Based Study
Source: J Clin Med. 2025 Apr 7;14(7):2524. doi: 10.3390/jcm14072524 (PMC11989609; doi:10.3390/jcm14072524)
Supplement: Supplementary file 1 [file jcm-14-02524-s001.zip › Supplementary Material S1.pdf]

KOMISJA BIOETYCZNA

Szczecin, dn. 20.01.2025 r.

KB.006.001/2025

Eryk Prajwos
Zakład Stomatologii Zintegrowanej
PUM

W związku ze zgłoszeniem do Komisji Bioetycznej Pomorskiego Uniwersytetu Medycznego w Szczecinie opisu badania pt.: *„Analiza bólu oraz przesuwania zębów w pierwszej fazie leczenia aparatami cienkołukowymi"* uprzejmie informuję, że przedmiotowe badanie nie wymaga opinii Komisji Bioetycznej.

Z poważaniem

PRZEWODNICZĄCY
Komisji Bioetycznej
Pomorskie[illegible] Uniwersytetu Medycznego
[illegible]ie

prof. dr hab. n. med. Marek Droździk
