# Supplementary material for: Pain and Tooth Movement During Orthodontic Leveling and Alignment—A Questionnaire-Based Study
Source: J Clin Med. 2025 Apr 7;14(7):2524. doi: 10.3390/jcm14072524 (PMC11989609; doi:10.3390/jcm14072524)
Supplement: Supplementary file 1 [file jcm-14-02524-s001.zip › Supplementary Material S2.pdf]

Ankieta na temat bólu oraz przesuwania zębów po założeniu aparatu stałego

Kryteria włączenia:

- **Wiek: 18–50 lat**
- **Pacjenci w trakcie leczenia aparatem stałym**
- **Okres od założenia aparatu: 0–6 miesięcy**
- **Ogólny stan zdrowia: dobry**

Zadane pytania:

1. Jaka jest Pana/Pani płeć?

a. Mężczyzna
b. Kobieta
c. Inne

2. Jaki jest Pana/Pani wiek?

a. 18-24
b. 25-34
c. 35-44
d. 45-50

3. Jakie jest Pana/Pani miejsce zamieszkania?

a. Wieś
b. Miasto do 50 tyś. mieszkańców
c. Miasto od 50 do 100 tyś. mieszkańców
d. Miasto od 100 do 500 tyś. mieszkańców
e. Miasto powyżej 500 tyś mieszkańców

4. Jakie jest Pana/Pani wykształcenie?

a. Podstawowe
b. Gimnazjalne
c. Średnie
d. Wyższe
e. Inne

5. Od jakiego czasu ma Pan/Pani założony aparat stały?

a. Mniej niż miesiąc
b. 1-2 miesiące
c. 3-4 miesiące
d. 5-6 miesięcy

6. Na który łuk miał Pan/Pani założony aparat?

a. Tylko górne zęby
b. Tylko dolne zęby
c. Górne i dolne zęby w tym samym czasie
d. Najpierw górne potem dolne zęby
e. Najpierw dolne potem górne zęby

7. Jaki był najmocniejszy ból jaki Pan/Pani doświadczył/a po założeniu aparatu? (użyj skali 1-100)

8. Jaka była charakterystyka tego bólu? (pytanie wielokrotnego wyboru)

a. Ból pojawiający się nagle
b. Ból znikający nagle
c. Ból stopniowo narastający
d. Ból stopniowo malejący
e. Stały ból o małym nasileniu
f. Stały ból o dużym nasileniu

9. Po jakim czasie od założenia aparatu zaczął się ból?

a. Od razu
b. Po 2-3h
c. Po 12h
d. Następnego dnia

10. Kiedy ból po założeniu aparatu był najbardziej intensywny?

a. Od razu
b. Po 1 godzinie
c. Po 6 godzinach
d. Następnego dnia

11. Czy brał Pan/Pani jakieś leki po założeniu aparatu?

a. Tak
b. Nie

12. Jakie leki stosował Pan/Pani po założeniu aparatu? (pytanie wielokrotnego wyboru)

a. Ibuprofen
b. Paracetamol
c. Inne

13. Czy ból nasilał się pod wpływem bodźców zewnętrznych?

a. Tak
b. Nie
c. Nie wiem

14. Jakie bodźce zwiększały ból?

a. Nagryzanie
b. Ciepło
c. Zimno
d. Inne

15. Które zęby były najbardziej bolesne? (pytanie wielokrotnego wyboru)

a. Górne siekacze
b. Dolne siekacze
c. Górne kły
d. Dolne kły
e. Górne przedtrzonowce i trzonowce
f. Dolne przedtrzonowce i trzonowce

16. Czy uważa Pan/Pani, że twoje zęby zaczęły się przesuwać od razu po założeniu aparatu?

a. Tak
b. Nie
c. Nie wiem

17. Które górne zęby zaczęły się przesuwać jako pierwsze? (pytanie wielokrotnego wyboru)

a. Siekacze
b. Kły
c. Przedtrzonowce
d. Trzonowce

18. Które dolne zęby zaczęły się przesuwać jako pierwsze? (pytanie wielokrotnego wyboru)

a. Siekacze
b. Kły
c. Przedtrzonowce
d. Trzonowce

19. Czy przyjmuje Pan/Pani regularnie jakieś leki?

a. Tak
b. Nie

20. Jakie leki bierze Pan/Pani na co dzień?

21. Czy zdiagnozowano u Pana/Pani jakieś choroby przewlekłe?

a. Tak
b. Nie

22. Jaką chorobę przewlekłą u Pana/Pani zdiagnozowano?

23. Czy zdiagnozowano u Pana/Pani jakiekolwiek zaburzenia psychoemocjonalne?

a. Tak
b. Nie

24. Jakie zaburzenia psychoemocjonalne u Pana/Pani zdiagnozowano?

English version:

1. What is your gender?

a. Male
b. Female
c. Other

2. What is your age?

a. 18–24
b. 25–34
c. 35–44
d. 45–50

3. What is your place of residence?

a. Town
b. City up to 50,000 inhabitants
c. City with 50,000 to 100,000 inhabitants
d. City with 100,000 to 500,000 inhabitants
e. City over 500,000 inhabitants

4. **What is your educational level?**

a. Primary education
b. Lower-secondary education
c. Secondary education
d. College education
e. Other

5. **For how long have you had the appliance on?**

a. Less than a month
b. 1–2 months
c. 3–4 months
d. 5–6 months

6. **Have you had braces applied to:**

a. Only the upper arch (top teeth)
b. Only the lower arch (bottom teeth)
c. Both upper and lower arches at the same time
d. First upper then lower arch
e. First lower then upper arch

7. **What was the highest level of pain that you experienced after having the braces? (answer in scale 1–100) (open answer question)**

8. **What was the dynamic nature of the pain? (multiple choice question)**

a. Sudden pain
b. Pain that stops suddenly
c. Gradually increasing pain
d. Gradually decreasing pain
e. Low-intensity constant pain
f. High-intensity constant pain

9. **How long after bonding braces did the pain start?**

a. Immediately
b. After 2–3 h
c. After 12 h
d. The next day
e.

10. **When was the pain most intense after getting the braces?**

a. Immediately
b. 1 h
c. 6 h
d. The next day

11. Had you taken any medications after getting braces?

a. Yes
b. No

12. What medications had you taken after getting the braces? (multiple choice question)

a. Ibuprofen
b. Paracetamol
c. Other

13. Did the pain increase in response to external stimuli?

a. Yes
b. No
c. I don't know

14. What external stimuli increased the pain? (multiple choice question)

a. Biting
b. Heat
c. Cold
d. Other

15. Which teeth experienced the most pain? (multiple choice question)

a. Upper incisors
b. Lower incisors
c. Upper canines
d. Lower canines
e. Upper premolars and molars
f. Lower premolars and molars

16. Do you think your teeth started to move immediately after getting the braces?

a. Yes
b. No
c. I don't know

17. Which teeth in the upper arch began to move first? (multiple choice question)

a. Incisors
b. Canines
c. Premolars
d. Molars

18. Which teeth in the lower arch began to move first? (multiple choice question)

a. Incisors
b. Canines
c. Premolars
d. Molars

19. Do you take any medications regularly?

a. Yes
b. No

20. What medications do you take on a regular basis? (open answer question)

21. Are you diagnosed with any chronic diseases?

a. Yes
b. No

22. What chronic diseases were you diagnosed with? (open answer question)

23. Are you diagnosed with any psychoemotional disorders?

a. Yes
b. No

24. What psychoemotional disorders are you diagnosed with? (open answer question)
